# Supplementary material for: The function of CozE proteins is linked to lipoteichoic acid biosynthesis in Staphylococcus aureus
Source: mBio. 2024 May 17;15(6):e01157-24. doi: 10.1128/mbio.01157-24 (PMC11237490; doi:10.1128/mbio.01157-24)
Supplement: Supplemental Material — Fig. S1-S14, captions to Movies S1 and S2, and Tables S1-S3. [file mbio.01157-24-s0001.pdf]

Supplemental Material

The function of CozE proteins is linked to lipoteichoic acid biosynthesis in *Staphylococcus aureus*

Maria Disen Barbuti[1], Elisabeth Lambert[2], Ine Storaker Myrbråten[1], Adrien Ducret[3], Gro Anita Stamsås[1], Linus Wilhelm[3], Xue Liu[4,5], Zhian Salehian[1], Jan-Willem Veening[5], Daniel Straume[1], Christophe Grangeasse[3], Camilo Perez[2,6] and Morten Kjos[1,*]

[1] Faculty of Chemistry, Biotechnology and Food Science, Norwegian University of Life Sciences, Ås, Norway.

[2] Biozentrum, University of Basel, Basel, Switzerland

[3] Molecular Microbiology and Structural Biochemistry, CNRS UM 5086, Université de Lyon, Lyon, France.

[4] Department of Pathogen, Biology, International Cancer Center, Shenzhen University Medical School, Shenzhen, Guangdong, 518055, China.

[5] Department of Fundamental Microbiology, University of Lausanne, Lausanne, Switzerland.

[6] Department of Biochemistry and Molecular Biology, University of Georgia, Athens, GA, United States.

[*] Correspondence to: morten.kjos@nmbu.no

Supplemental figures

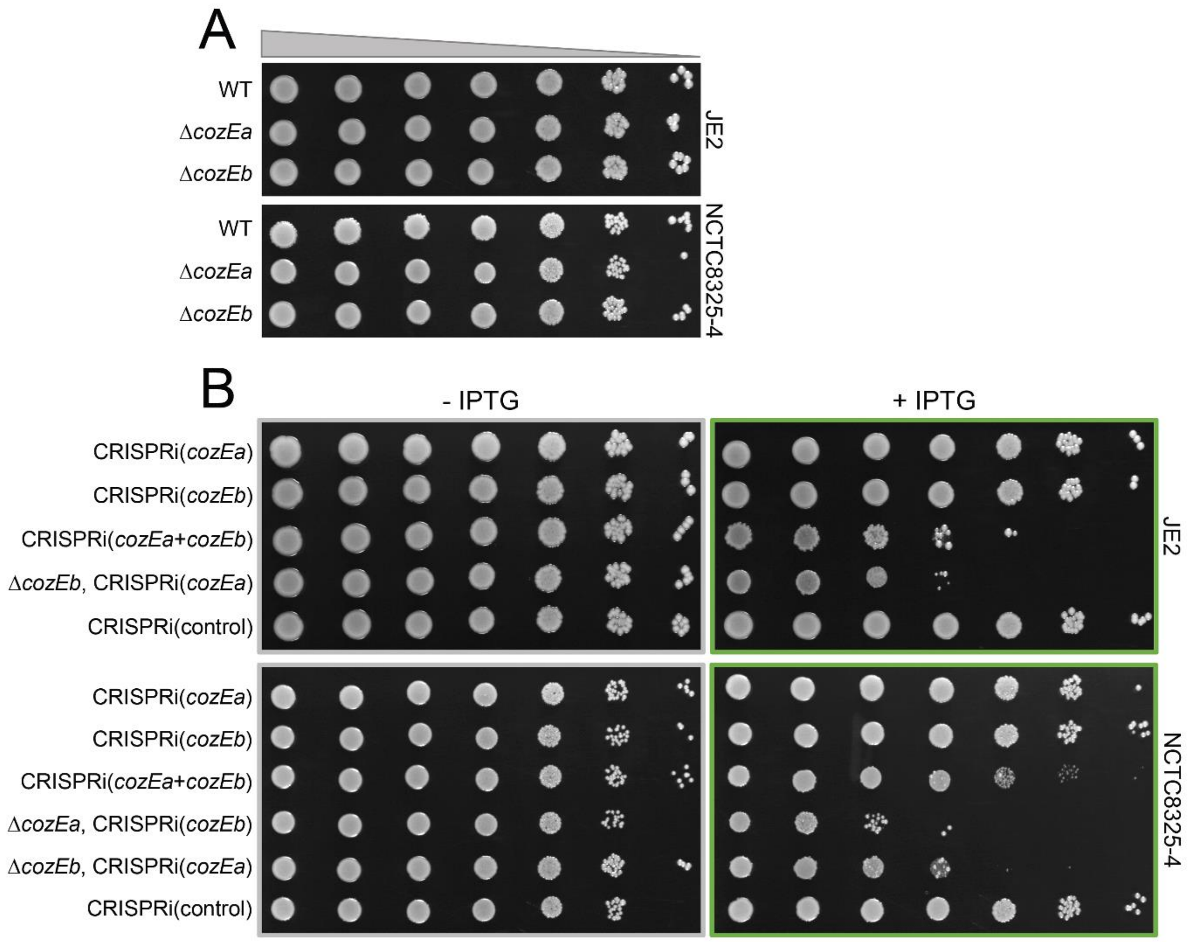

Fig. S1. Growth of single and double *cozE* mutants in *S. aureus* JE2 and NCTC8325-4.
(**A**) Growth on solid medium of wild-type, Δ*cozEa*, and Δ*cozEb* in *S. aureus* JE2 (MDB37, MDB38, and MDB10) and NCTC8325-4 (MDB1, MDB2, and MDB3). 10-fold dilution series, made from overnight cultures, were spotted onto agar plates. (**B**) Growth on solid medium of single and double *cozE* knockdown strains in *S. aureus* JE2 (MDB17, MDB18, MDB19, MDB21, and MDB44) and NCTC8325-4 (MDB14, MDB15, MDB13, MDB11, MDB12, and MM75). 10-fold dilution series, made from noninduced overnight cultures, were spotted onto agar plates with and without IPTG, as indicated. Strains carrying a non-targeting sgRNA were used as controls.

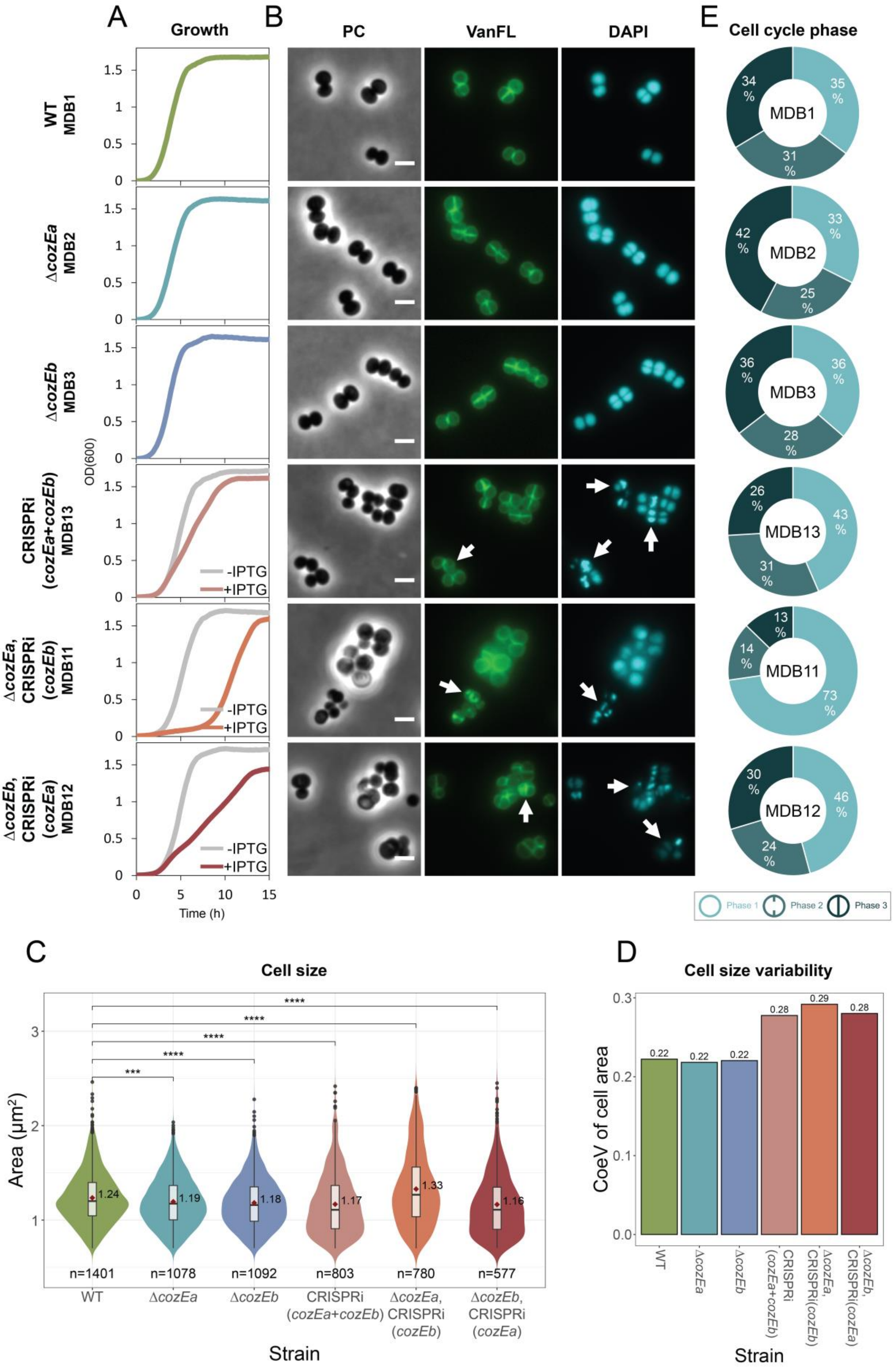
A
Growth
B
PC
VanFL
DAPI
E
Cell cycle phase
WT MDB1
ΔcozEa MDB2
ΔcozEb MDB3
CRISPRi (cozEa+cozEb) MDB13
ΔcozEa, CRISPRi (cozEb) MDB11
ΔcozEb, CRISPRi (cozEa) MDB12
OD(600)
Time (h)
-IPTG
+IPTG
MDB1
MDB2
MDB3
MDB13
MDB11
MDB12
Phase 1
Phase 2
Phase 3
C
Cell size
Area (μm²)
Strain
n=1401
n=1078
n=1092
n=803
n=780
n=577
WT
ΔcozEa
ΔcozEb
CRISPRi (cozEa+cozEb)
ΔcozEa, CRISPRi (cozEb)
ΔcozEb, CRISPRi (cozEa)
D
Cell size variability
CoeV of cell area
Strain

Fig. S2. Morphological and cell cycle analysis of single and double *cozE* mutants in *S. aureus* NCTC8325-4.

(**A**) Growth curves of NCTC8325-4 wild-type (MDB1), Δ*cozEa* (MDB2) and Δ*cozEb* (MDB3), as well as of a CRISPRi double knockdown strain (CRISPRi(*cozEa*+*cozEb*), MDB13) and combined knockout/knockdown strains (Δ*cozEa*, CRISPRi(*cozEb*), MDB11, and Δ*cozEb*, CRISPRi(*cozEa*), MDB12) in BHI medium at 37°C. The graphs represent averages from triplicate measurements. The CRISPRi-strains were grown with and without IPTG, as indicated. (**B**) Micrographs of the same strains as in (A) showing phase contrast (PC) and fluorescence microscopy of cells stained with the cell wall label VanFL and the nucleoid label DAPI. CRISPRi strains were grown in medium with IPTG to induce the CRISPRi system. White arrows point to cells with perturbed septum formation and abnormal nucleoid staining. The scale bars are 2 µm. (**C**) Violin plots of the cell areas (in μm^2) of NCTC8325-4 wild-type (1.24 ± 0.27 μm^2), Δ*cozEa* (1.19 ± 0.26 μm^2), Δ*cozEb* (1.18 ± 0.27 μm^2), MDB13 (1.17 ± 0.32 μm^2), MDB11 (1.33 ± 0.39 μm^2), and MDB12 (1.16 ± 0.33 μm^2), determined using MicrobeJ. Significant differences between the strains are indicted with asterisks (* indicates a P-value of < 0.05, ** indicates a P-value of < 0.01, and *** indicates a P-value of < 0.001, derived from a Mann-Whitney test). The number of cells analyzed for each strain is indicated in the figure. (**D**) Coefficient of variance (CoeV) of cell size for each strain based on the data provided in (C), showing the relative dispersion of cell area for each strain around their perspective means (CoeV = standard deviation of cell area / mean cell area). (**E**) Frequency of cells in each of the three cell cycle phases for NCTC8325-4 wild-type, Δ*cozEa*, Δ*cozEb*, MDB13, MDB11, and MDB12. See Fig. 1D in the main article for a schematic overview of the different phases analyzed. The distributions were obtained by manually counting the different cell cycle phases of 100-150 randomly selected VanFL stained cells from each strain.

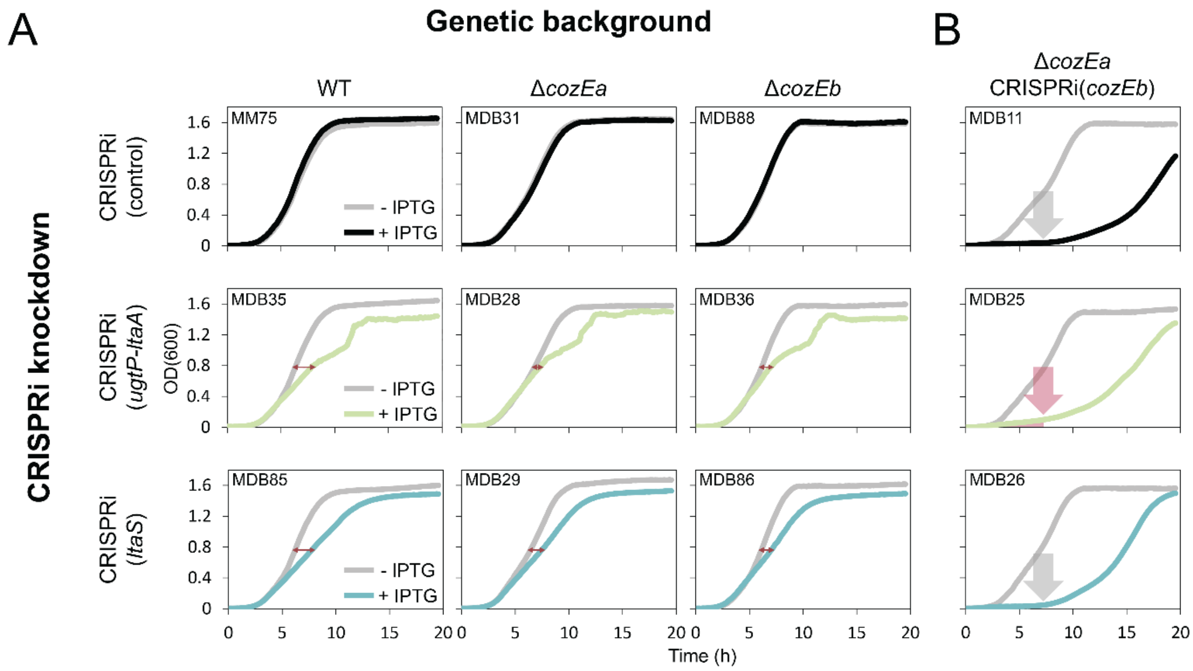

Fig. S3. **Synthetic genetic relationships between *cozE* genes and genes involved in LTA biosynthesis.**
(**A**) Growth assays to identify possible genetic interactions between *cozEa* or *cozEb* and the LTA biosynthetic pathway. *ugtP-ltaA* or *ltaS* was knocked down by CRISPRi in either a NCTC8325-4 wild-type, Δ*cozEa*, or Δ*cozEb* background. The CRISPRi-strains were grown with and without IPTG, as indicated. The length of the red arrows illustrates the growth differences observed between the genetic backgrounds, wild-type, Δ*cozEa*, and Δ*cozEb*, when UgtP-LtaA or LtaS were depleted. (**B**) Growth assays to identify possible genetic interactions between *cozEab* and the LTA biosynthetic pathway. *ugtP-ltaA* or *ltaS* was knocked down along with *cozEb* in a Δ*cozEa* background. The CRISPRi-strains were grown with and without IPTG, as indicated by the colors. The red arrow points to the slight growth alleviation observed when *ugtP-ltaA* was knocked down together with *cozEb* in the Δ*cozEa* background (MDB25). In both (A) and (B), the graphs represent averages from triplicate measurements.

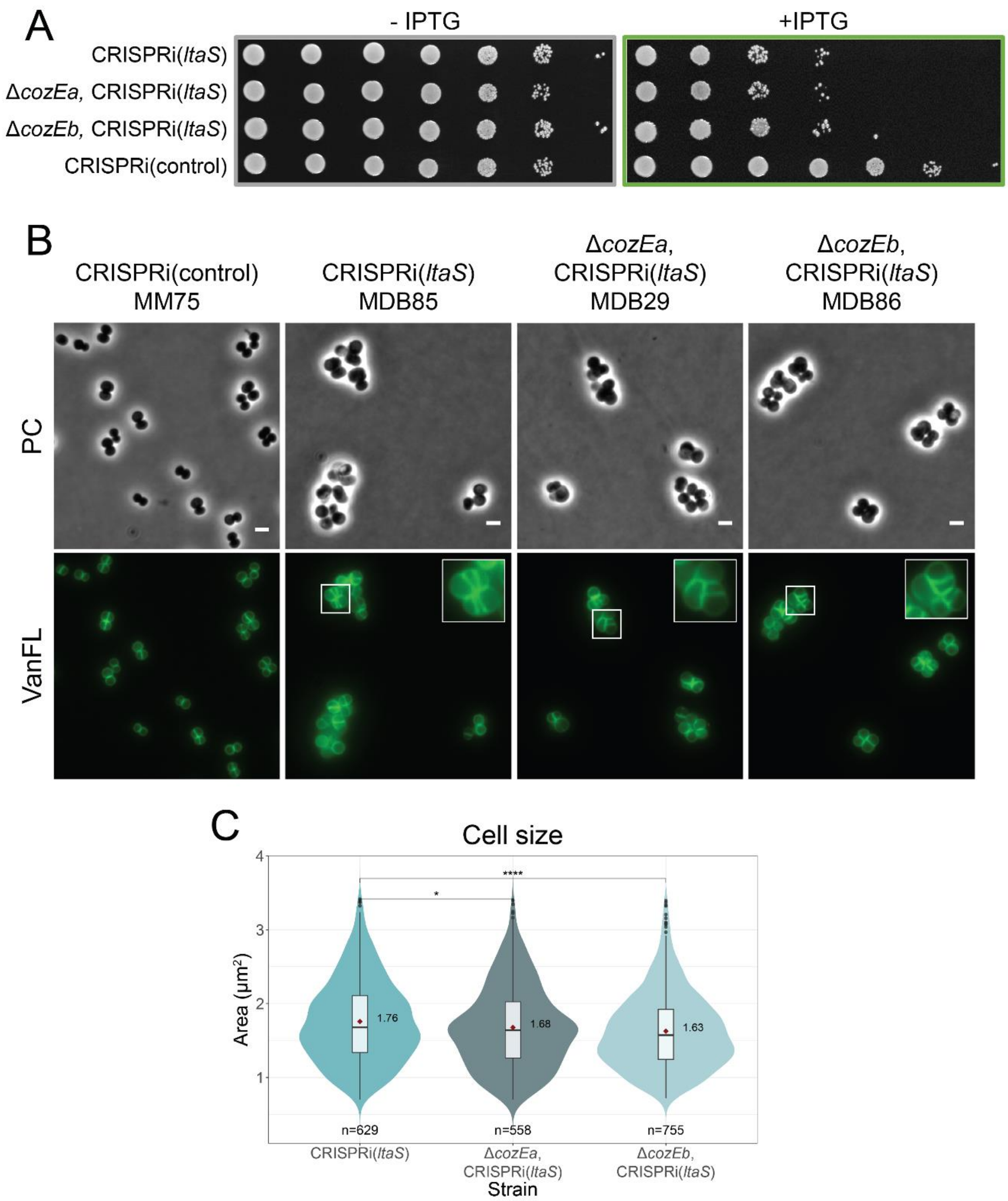

Fig. S4. LtaS depletion in wild-type, Δ*cozEa*, and Δ*cozEb S. aureus* NCTC8325-4 cells.
(**A**) Growth on solid medium of LtaS depleted *S. aureus* NCTC8325-4 cells; CRISPRi(*ltaS*) (MDB85), Δ*cozEa*, CRISPRi(*ltaS*) (MDB29), and Δ*cozEb*, CRISPRi(*ltaS*) (MDB86). In addition to a strain carrying a non-targeting sgRNA (MM75) used as a control. 10-fold dilution series, made from noninduced overnight cultures, were spotted onto agar plates with and without IPTG, as indicated. (**B**) Phase contrast (PC) and VanFL staining micrographs of the same strains as in (A). The cells were grown in the presence of IPTG for induction of the CRISPRi system. The scale bars are 2 µm. (**C**) Violin plots of the cell areas (in μm^2) of NCTC8325-4 CRISPRi(*ltaS*) (1.76 ± 0.55 μm^2), Δ*cozEa*, CRISPRi(*ltaS*) (1.68 ± 0.55), and Δ*cozEb*, CRISPRi(*ltaS*) (1.63 ± 0.51 μm^2), determined using MicrobeJ. Significant differences between the strains are indicted with asterisks (* indicates a P-value of < 0.05, ** indicates a P-value of < 0.01, and *** indicates a P-value of < 0.001, derived from a Mann-Whitney test). The number of cells analyzed for each strain is indicated in the figure.

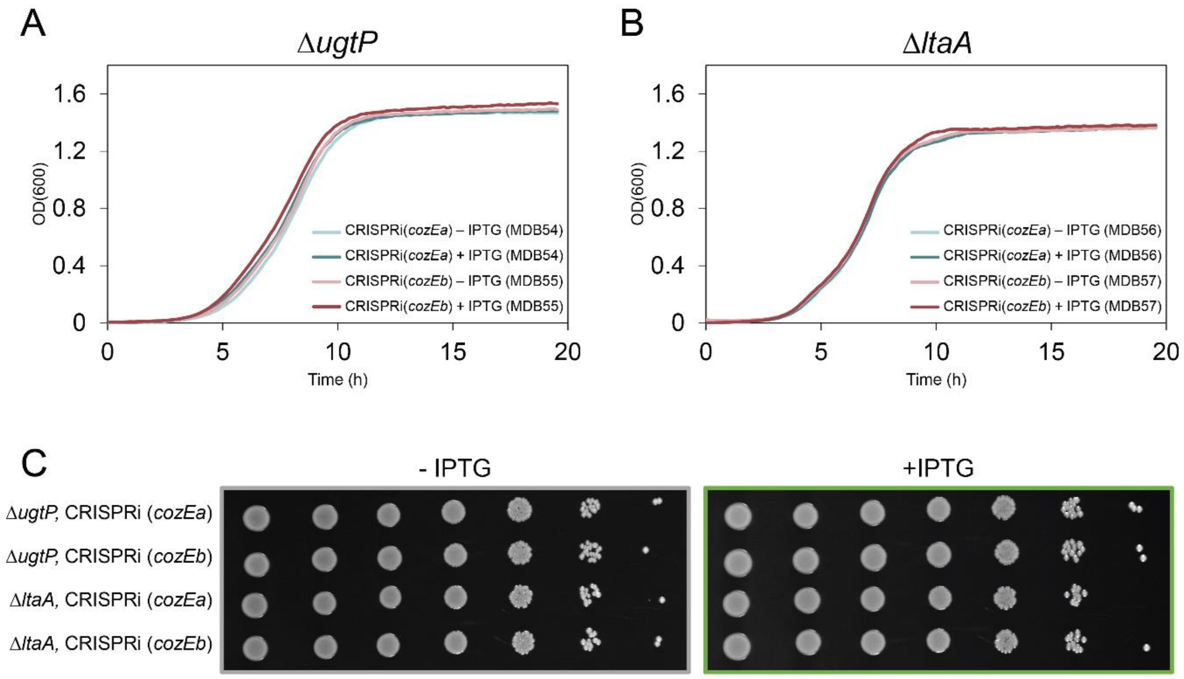

Fig. S5. Single CozE depletion in *S. aureus* JE2 Δ*ugtP* and Δ*ltaA*.
Growth curves of JE2 (**A**) Δ*ugtP* and (**B**) Δ*ltaA* with individual depletion of CozEa or CozEb in BHI medium at 37°C. The graphs represent averages from triplicate measurements. The CRISPRi-strains were grown with and without IPTG, as indicated. (**C**) Additionally, growth of the JE2 Δ*ugtP* and Δ*ltaA* strains with single CozE depletion on solid medium. 10-fold dilution series, made from noninduced overnight cultures, were spotted onto agar plates with and without IPTG, as indicated.

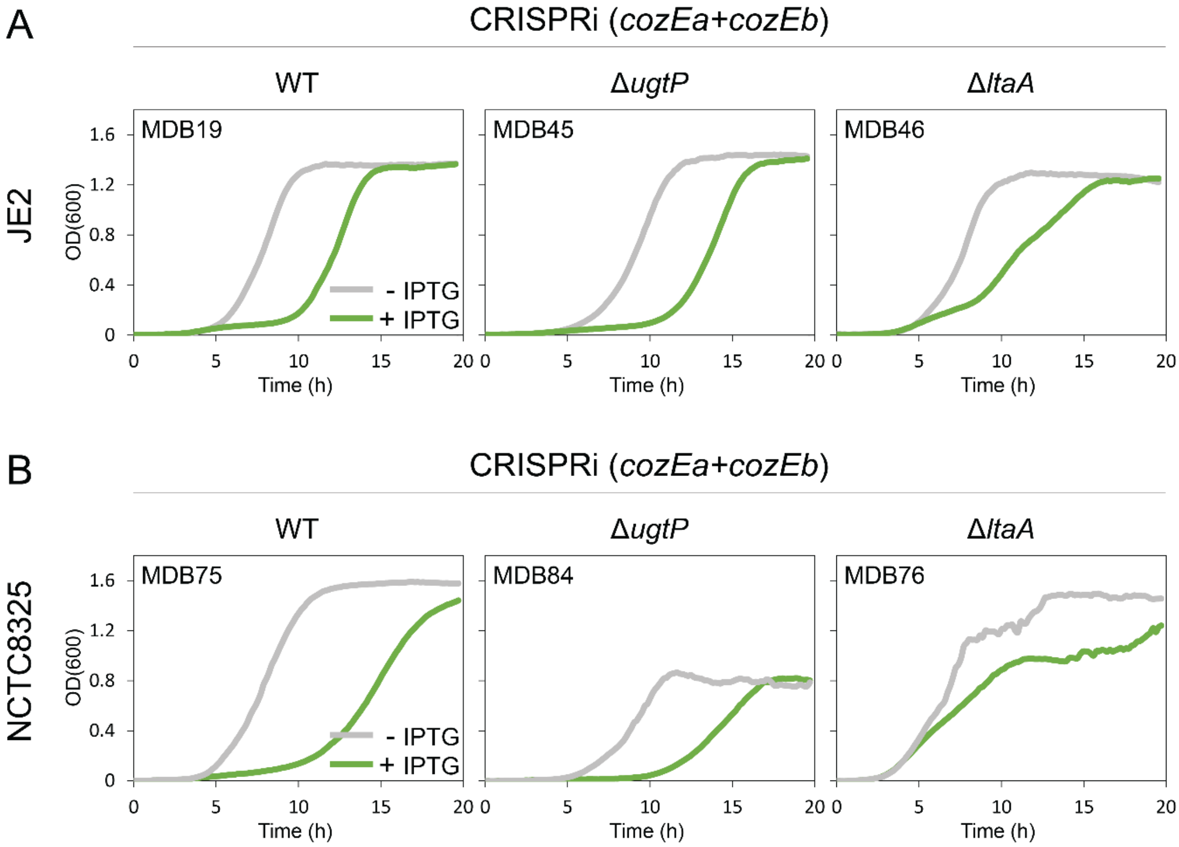

Fig. S6. **Growth of wild-type, Δ*ugtP*, and Δ*ltaA* with double CozE depletion in *S. aureus* JE2 and NCTC8325, using 500 µM IPTG for maximum depletion.**

Growth of wild-type, Δ*ugtP*, and Δ*ltaA* with double *cozE* knockdown in (**A**) JE2 (MDB19, MDB45, and MDB46) and (**B**) NCTC8325 (MDB75, MDB84, MDB76) in liquid cultures. Cells were grown in the presence or absence of 500 µM IPTG, as indicated by the colors. The graphs represent averages from triplicate measurements.

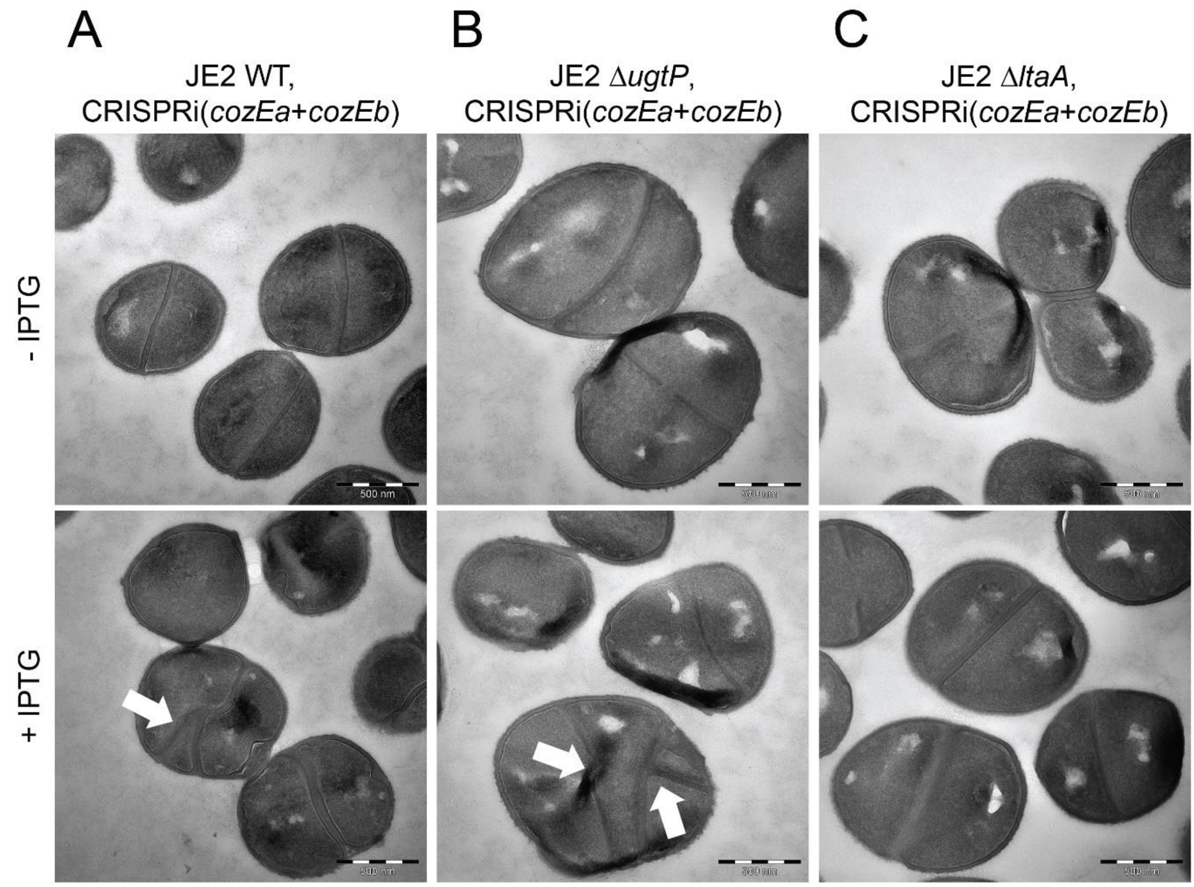

Fig. S7. TEM analysis of *S. aureus* JE2 wild-type, Δ*ugtP*, and Δ*ltaA* mutants with double CozE depletion. TEM micrographs of JE2 (**A**) wild-type, (**B**) Δ*ugtP*, and (**C**) Δ*ltaA* cells with uninduced or induced depletion of CozEa and CozEb. White arrows point to cells with aberrant septum formation. The scale bars are 500 nm.

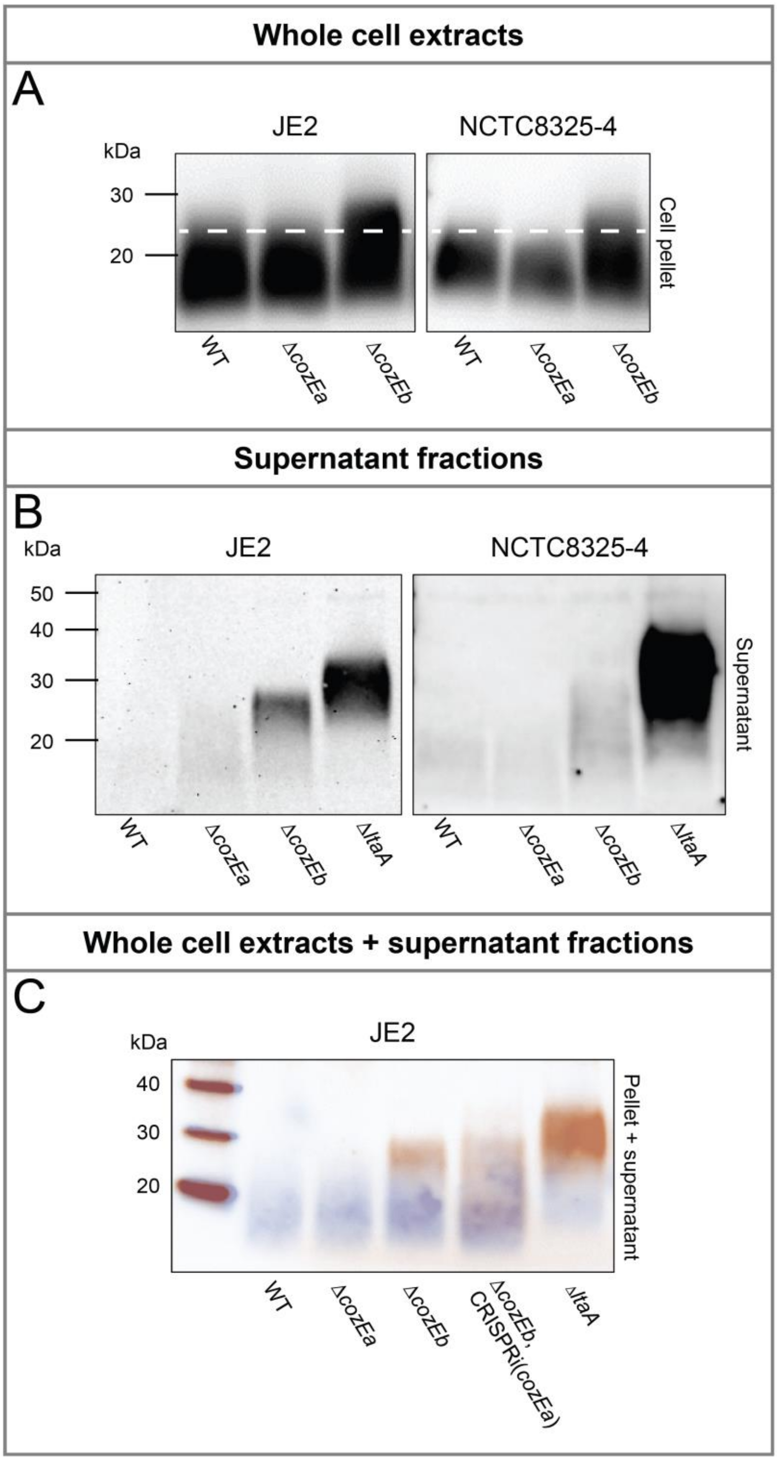

Fig. S8. Characterization of LTA polymer length and stability in single and double *cozE* mutants.
LTA polymers were detected in whole cell extracts, (A) and (C), or in supernatant fractions, (B) and (C), by immunoblotting with an anti-LTA antibody. (**A**) Immunoblots of wild-type, Δ*cozEa*, and Δ*cozEb* in JE2 (MDB37, MDB38, and MDB10) and NCTC8325-4 (MDB1, MDB2, and MDB3). (**B**) Immunoblots of wild-type, Δ*cozEa*, Δ*cozEb*, and Δ*ltaA* in JE2 (MDB9, MDB38, MDB10, and MDB40) and NCTC8325-4 (MDB1, MDB2, MDB3, and MDB69). (**C**) Merged immunoblots of JE2 wild-type (MDB9), Δ*cozEa* (MDB38), Δ*cozEb* (MDB10), a double *cozE* mutant (MDB21), and a positive control strain (MDB40), where the LTA detected in the cell pellets are colored blue while the LTA detected in the supernatants are colored orange to illustrate the differences in LTA polymer lengths.

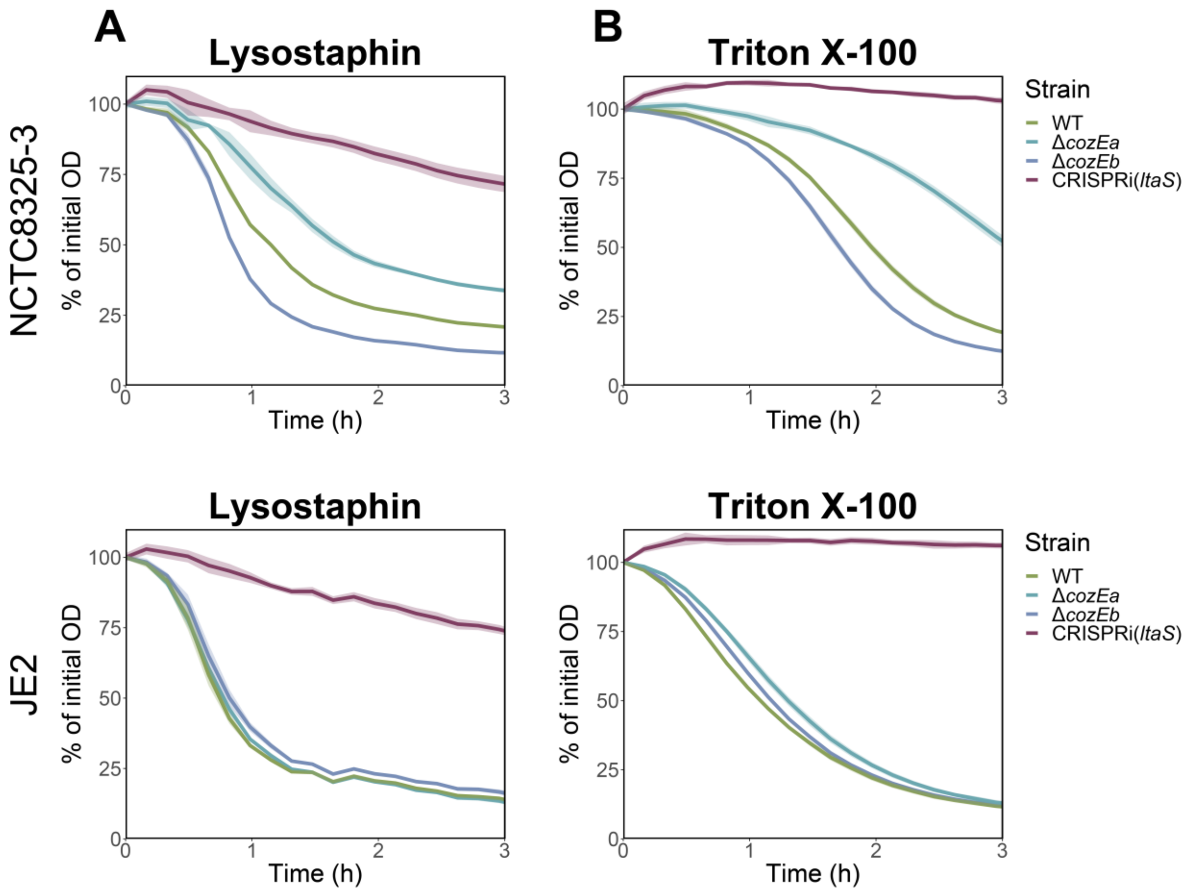

Fig. S9. Autolytic behavior of single *cozE* mutants.
Autolysis of wild-type, Δ*cozEa*, Δ*cozEb*, and CRISPRi(*ltaS*) in *S. aureus* NCTC8325-4 (strains MDB1, MDB2, MDB3, and MDB85) and JE2 (strains MDB9, MDB38, MDB10, and MDB375) measured in PBS buffer containing (**A**) lysostaphin (100 ng/ml) or (**B**) Triton X-100 (0.05%). For all plots, the data are presented as percentage of initial OD_{600}, with averages and standard deviations from four technical replicates.

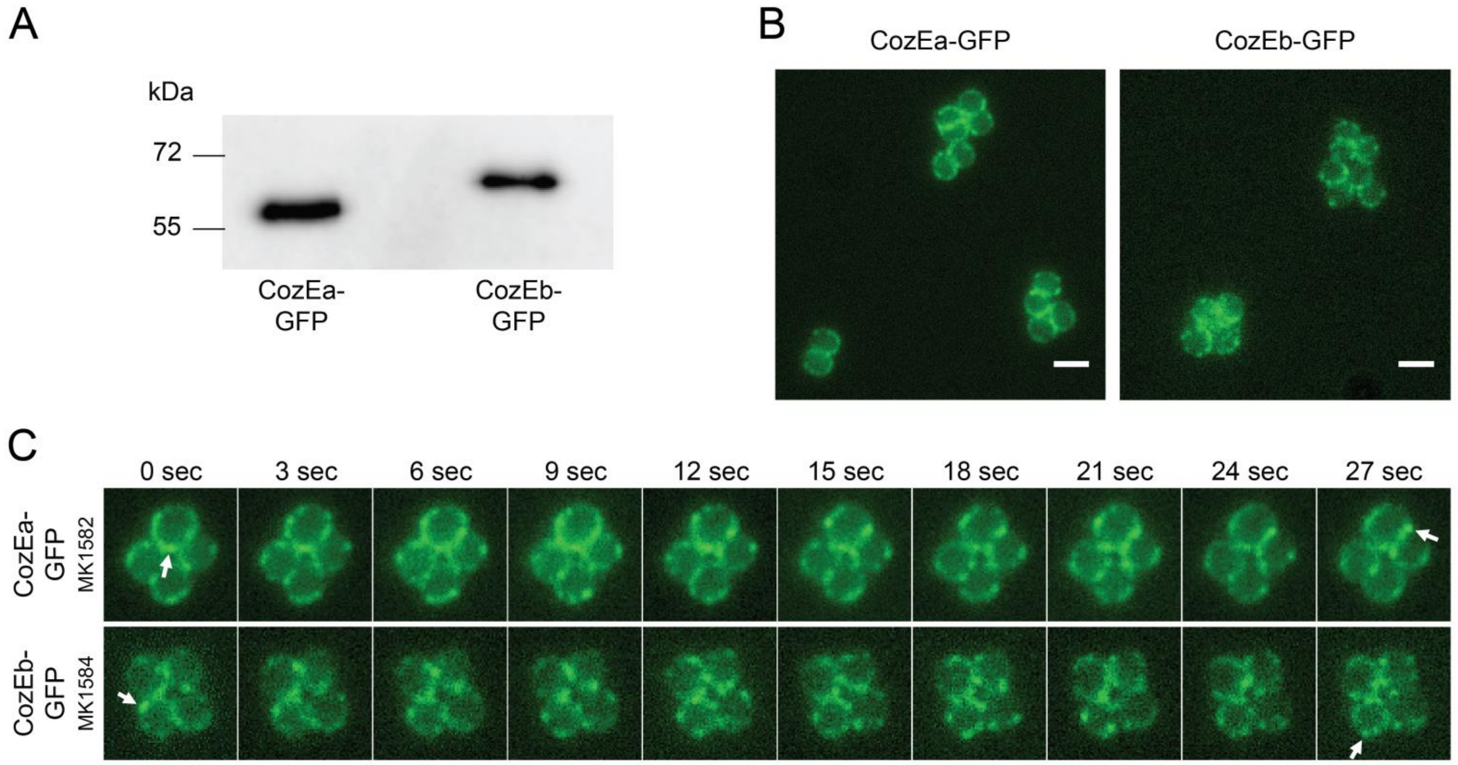

Fig. S10. Subcellular localization of CozEa and CozEb.
(**A**) The relative expression of CozEa and CozEb is indicated by the band density of CozEa-GFP and CozEb-GFP (from MK1582 and MK1584, respectively, which were incubated until they reached an OD_{600} of ~0.4 and subsequently normalized) in an immunoblot assay using an anti-GFP antibody. (**B**) The subcellular localization of CozEa and CozEb analyzed by florescent microscopy of MK1582 and MK1584, respectively. The scale bars are 2 µm. (**C**) The movement of the CozE proteins were analyzed by time-lapse florescent microscopy of MK1582 (with a *cozEa-gfp* fusion) and MK1584 (with a *cozEb-gfp* fusion). Images were captured every third second (x10), as indicated in the figure. White arrows highlight the membrane movement of CozEa-GFP and CozEb-GFP, as the signals pointed to in the initial images are no longer present at the same location in the membrane after 27 seconds. The dynamic spatiotemporal localization of CozEa and CozEb is further depicted in **Movie S1** and **S2**, respectively.

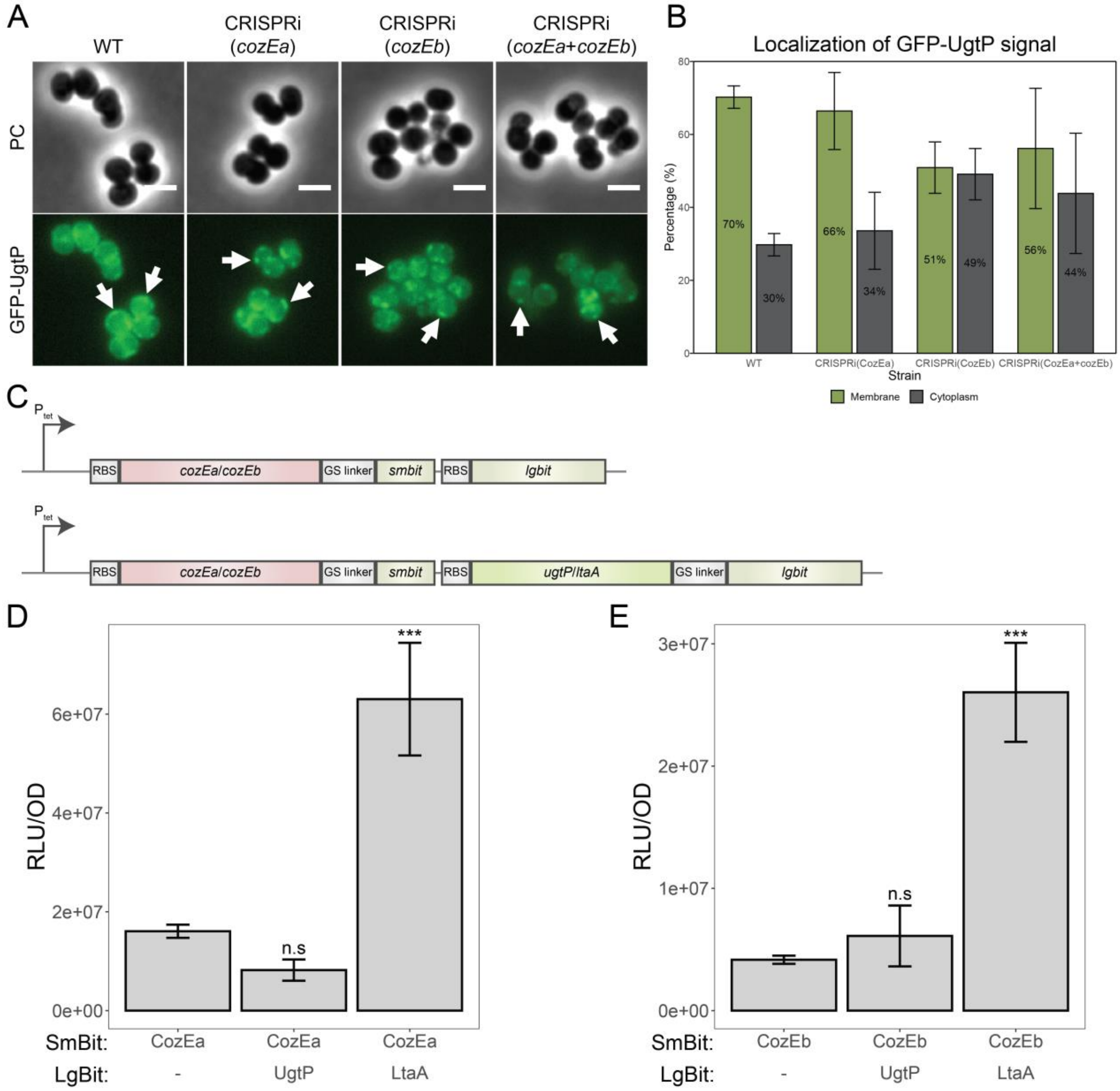

Fig. S11. Analysis of direct interaction between CozE and UgtP or LtaA.
(**A**) Localization of GFP-UgtP in NCTC8325-4 wild-type (MDB77), as well as cells depleted of CozEa (MDB89), CozEb (MDB90), or both CozE proteins (MDB79). Phase contrast- and fluorescence images are shown. Arrows point to spots with membrane-localized GFP-UgtP. The scale bars are 2 µm. (**B**) Quantification of GFP-UgtP subcellular localization in the strains shown in (A). The distributions were obtained by manually counting the localization pattern of 200-250 randomly selected cells from each strain. The data presented are the averages from three independent experiments. (**C**) Schematic illustration of the split luciferase plasmids constructed in this work to assess pairwise protein–protein interactions *in vivo* in *S. aureus*. The proteins of interest (CozE, UgtP, and LtaA) were fused to either a large (LgBit) or small (SmBit) luciferase subunit, which upon interaction form an active luciferase enzyme that generate a bright luminescent signal. Vectors encoding CozE fused to SmBit and LgBiT, not part of a fusion protein, were used as negative controls (pAF256 vectors were used as controls in this work because they are found to have a higher occurrence of non-specific luciferase activity than pAF257 and pAF262 in *S. aureus* (1)). Split luciferase assays identifying potential protein-protein interactions between (**D**) CozEa and UgtP/LtaA and (**E**) CozEb and UgtP/LtaA. The average RLU/OD_{600} of four replicates are shown, with error bars indicating the standard deviation from the mean. Positive interactions were determined based of the negative controls, by performing a one-way ANOVA followed by Tukey's Honest Significant Difference (HSD) test (n.s indicates a P-value of > 0.05, while *** indicates a P-value of < 0.001). The assays revealed that neither CozEa nor CozEb directly interact with UgtP, while both CozE proteins interact with LtaA.

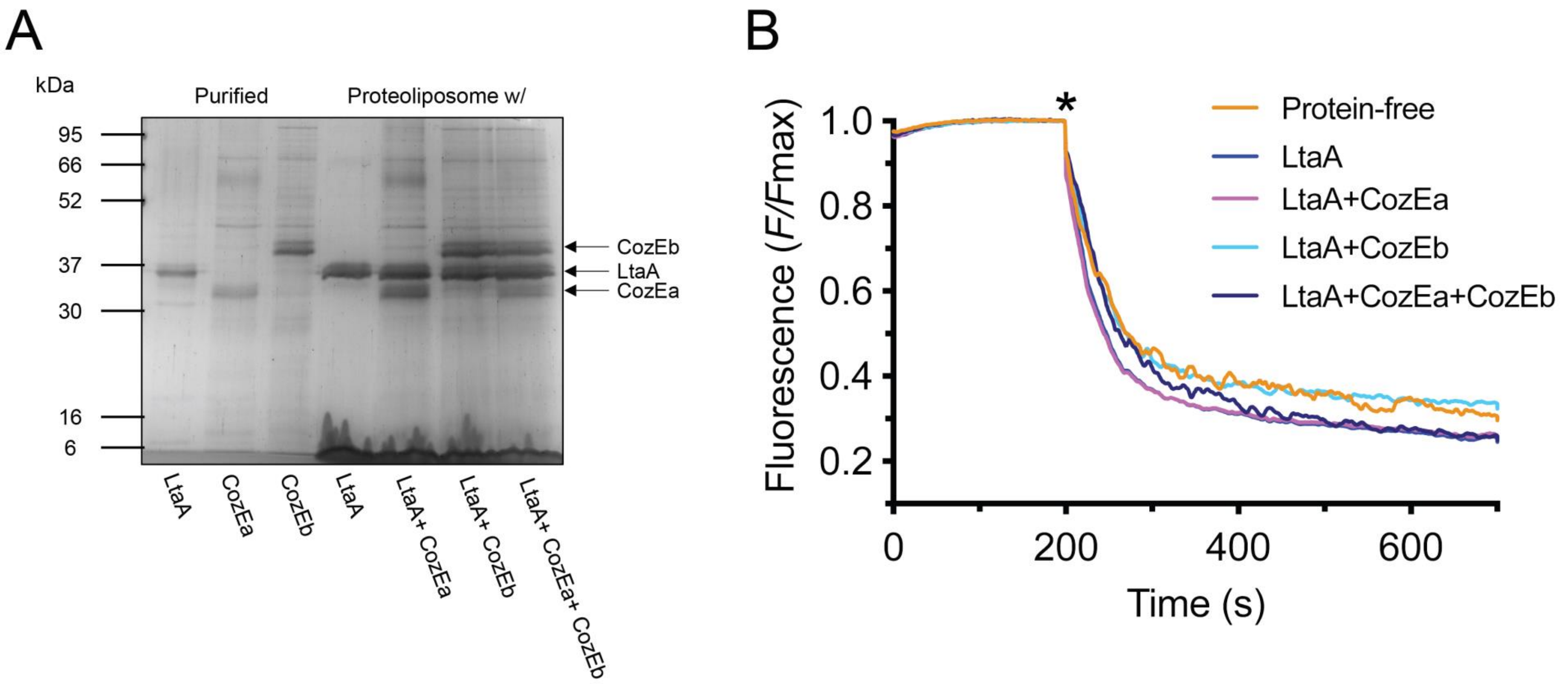

Fig. S12. LtaA-catalyzed Glc_2DAG flipping in the presence of CozE proteins.

(**A**) SDS-PAGE of purified (1) LtaA (44.6 kDa), (2) CozEa (40.1 kDa), and (3) CozEb (45.1 kDa), in addition to proteoliposomes with (1) LtaA, (2) LtaA and CozEa, (3) LtaA and CozEb, and (4) LtaA, CozEa, and CozEb used in the Glc_2DAG flipping experiment. Arrows point at LtaA, CozEa, and CozEb. (**B**) Representative traces of proteoliposomes containing LtaA and LtaA together with CozE proteins and a protein-free liposome (n = 3). The asterisk marks addition of dithionite. F corresponds to the fluorescence intensity measured for each time point. Fmax is the average fluorescence measured during the first 200 seconds of the experiment.

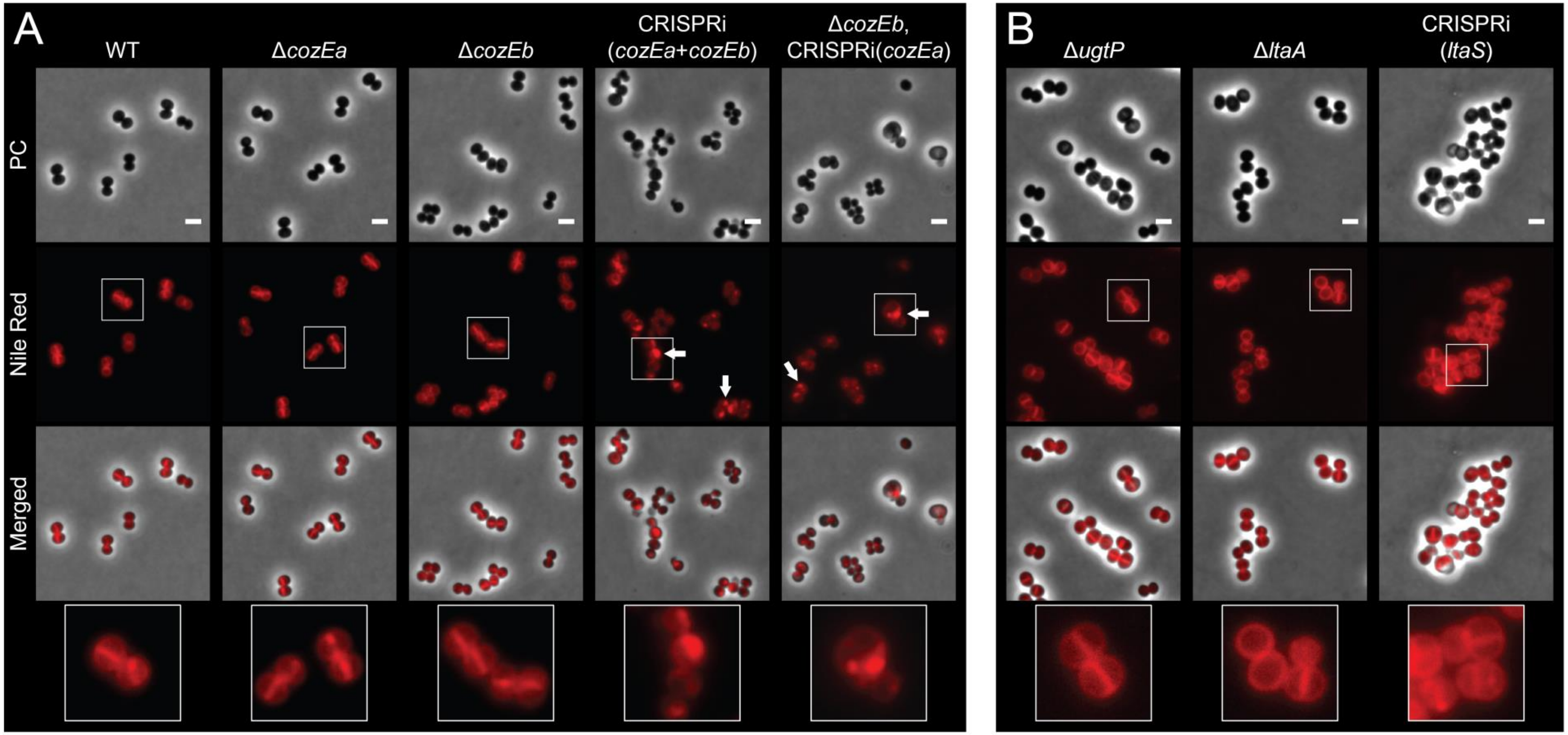

Fig. S13. Microscopic analysis of membrane staining in *cozE* mutants and LTA biosynthesis mutants.
Phase contrast (PC) and Nile Red staining micrographs of *S. aureus* JE2 (**A**) *cozE* mutants; wild-type (MDB9), Δ*cozEa*::Tn (MDB38), Δ*cozEb*::Tn (MDB10), a CRISPRi double knockdown strain (CRISPRi(*cozEa*+*cozEb*), MDB19) and a combined knockout/knockdown strain (Δ*cozEb*+CRISPRi(*cozEa*), MDB21) and (**B**) LTA biosynthesis mutants; Δ*ugtP*::Tn (MDB39), Δ*ltaA*::Tn (MDB40) and a CRISPRi *ltaS* knockdown strain (MDB375). CRISPRi strains were grown in medium with IPTG to induce the CRISPRi-system. White arrows point to cells with perturbed membrane formation. The scale bars are 2 µm.

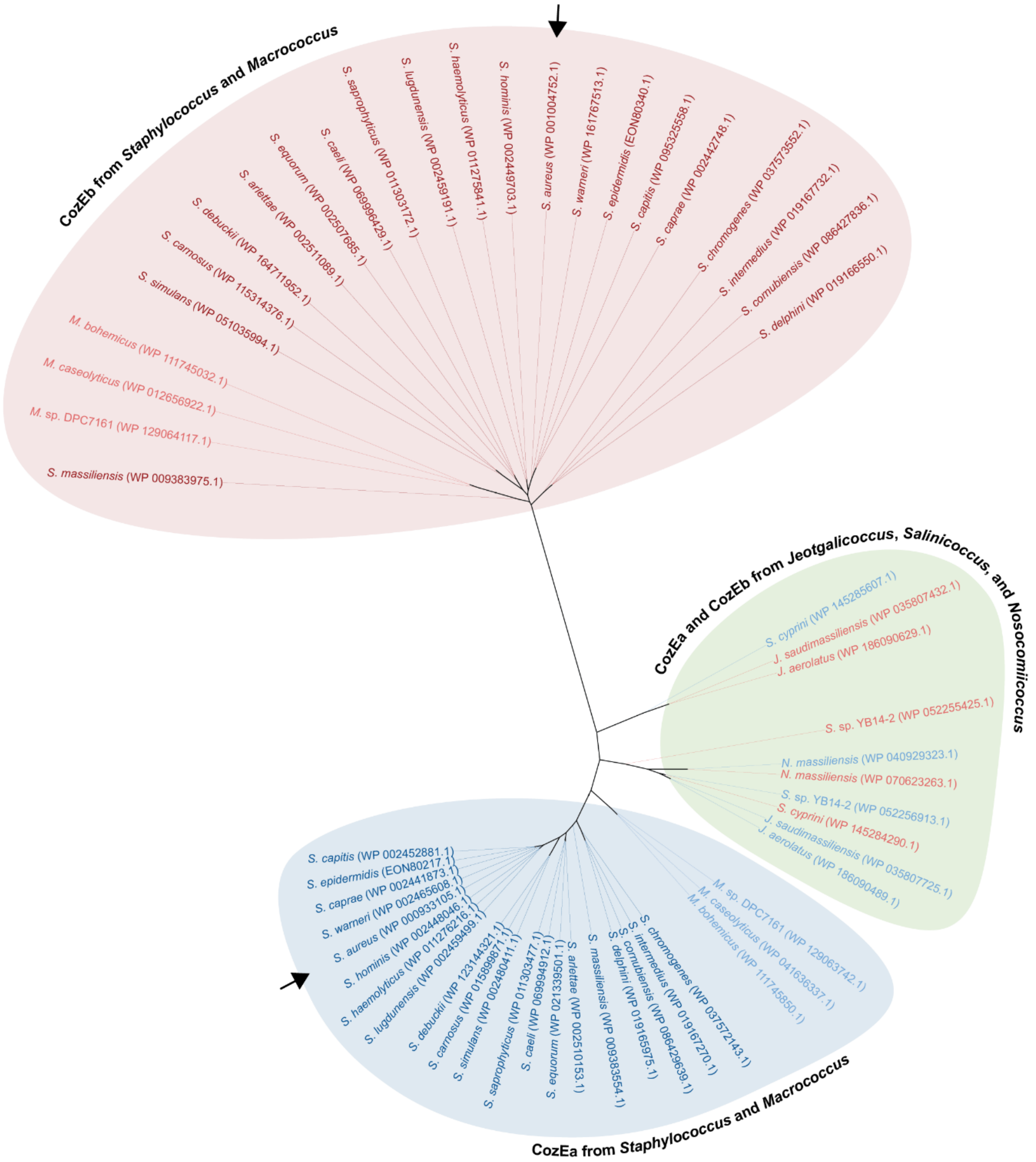

Fig. S14. Phylogenetic distribution of CozE proteins in the *Staphylococcaceae* family.
A maximum likelihood phylogenetic tree was constructed from a Clustal Omega sequence alignment of 56 CozE homologs from the *Staphylococcaceae* family, specifically *Staphylococcus*, *Macrococcus*, *Jeotgalicoccus*, *Salinicoccus*, and *Nosocomiicoccus*, using IQ-TREE. The phylogenetic tree was visualized and annotated using iTOL. The CozE proteins from species belonging to *Staphylococcus* and *Macrococcus* distributed into two phylogenetically separate subgroups; CozEa (marked in blue) and CozEb (marked in red). The CozE proteins from *Jeotgalicoccus*, *Salinicoccus*, and *Nosocomiicoccus* clustered together in another subgroup (marked in green), which is phylogenetically closer to the CozEa subgroup than the CozEb subgroup. Arrows point at the CozE proteins found in *S. aureus*.

Supplemental movies

Movie S1 (separate file). The dynamic spatiotemporal localization of GFP-tagged CozEa.
The movement of CozEa was analyzed by time-lapse fluorescent microscopy of MK1582, a NCTC8325-4 mutant with a *cozEa-gfp* fusion gene chromosomally integrated in its native locus. Images were captured every third second (x10), as indicated in the movie.

Movie S2 (separate file). The dynamic spatiotemporal localization of GFP-tagged CozEb.
The movement of CozEb was analyzed by time-lapse fluorescent microscopy of MK1584, a NCTC8325-4 mutant with a *cozEb-gfp* fusion gene chromosomally integrated in its native locus. Images were captured every third second (x10), as indicated in the movie.

Supplemental tables

Table S1. Strains and mutants used in this work.

Name	Genotype and characteristics[a]	Reference
***S. aureus* JE2**		
JE2/MDB9/ MDB37	Community acquired MRSA strain, derivative of USA300 LAC cured of plasmids	(2)
MDB38/ NE1270	JE2 Δ*cozEa*, ery^r	(2)
MDB10/ NE779	JE2 Δ*cozEb*, ery^r	(2)
MDB39/ NE1663	JE2 Δ*ugtP*, ery^r	(2)
MDB40/ NE462	JE2 Δ*ltaA*, ery^r	(2)
MDB16	JE2 carrying pLOW-dCas9_aad9, spc^r	This work
MDB20	MDB10 carrying pLOW-dCas9_aad9, spc^r	This work
MDB41	MDB39 carrying pLOW-dCas9_aad9, spc^r	This work
MDB42	MDB40 carrying pLOW-dCas9_aad9, spc^r	This work
MDB17	MDB16 carrying pCG248-sgRNA(*cozEa*), spc^r, cam^r	This work
MDB18	MDB16 carrying pCG248-sgRNA(*cozEb*), spc^r, cam^r	This work
MDB19	MDB16 carrying pCG248-sgRNA(*cozEa*+*cozEb*), spc^r, cam^r	This work
MDB375	MDB16 carrying pVL2336-sgRNA(*ltaS*), spc^r, cam^r	This work
MDB44	MDB16 carrying pCG248-sgRNA(*luc*), spc^r, cam^r	This work
MDB21	MDB20 carrying pCG248-sgRNA(*cozEa*+*cozEb*), spc^r, cam^r	This work
MDB45	MDB41 carrying pCG248-sgRNA(*cozEa*+*cozEb*), spc^r, cam^r	This work
MDB46	MDB42 carrying pCG248-sgRNA(*cozEa*+*cozEb*), spc^r, cam^r	This work
MDB54	MDB41 carrying pCG248-sgRNA(*cozEa*), spc^r, cam^r	This work
MDB55	MDB41 carrying pCG248-sgRNA(*cozEb*), spc^r, cam^r	This work
MDB56	MDB42 carrying pCG248-sgRNA(*cozEa*), spc^r, cam^r	This work
MDB57	MDB42 carrying pCG248-sgRNA(*cozEb*), spc^r, cam^r	This work
MDB59	MDB10 carrying pRAB11-*cozEa*, cam^r	This work
MDB60	MDB10 carrying pRAB11-*cozEb*, cam^r	This work
MDB176	MDB10 carrying pRAB11, cam^r	This work
***S. aureus* NCTC8325-4**		
NCTC8325-4/ MDB1	MSSA lab strain, derivative of NCTC8325 cured of prophages	(3)
MDB2	NCTC8325-4 Δ*cozEa*, spc^r	This work
MDB3	NCTC8325-4 Δ*cozEb*, spc^r	This work
MH225	NCTC8325-4 carrying pLOW-dCas9_extra_*lacO*, ery^r	Lab collection
MH223	MDB2 carrying pLOW-dCas9_extra_*lacO*, ery^r	This work
MH224	MDB3 carrying pLOW-dCas9_extra_*lacO*, ery^r	This work
MDB11	MH223 carrying pCG248-sgRNA(*cozEb*), ery^r, cam^r	This work
MDB12	MH224 carrying pCG248-sgRNA(*cozEa*), ery^r, cam^r	This work
MDB13	MH225 carrying pCG248-sgRNA(*cozEa*+*cozEb*), ery^r, cam^r	This work
MDB14	MH225 carrying pCG248-sgRNA(*cozEa*), ery^r, cam^r	This work
MDB15	MH225 carrying pCG248-sgRNA(*cozEb*), ery^r, cam^r	This work
MM75	MH225 carrying pVL2336-sgRNA(*luc*), ery^r, cam^r	Lab collection
MDB31	MH223 carrying pCG248-sgRNA(*luc*), ery^r, cam^r	This work
MDB88	MH224 carrying pCG248-sgRNA(*luc*), ery^r, cam^r	This work

MDB25	MH223 carrying pCG248-sgRNA(*cozEb+ugtP-ltaA*), ery^r, cam^r	This work
MDB26	MH223 carrying pCG248-sgRNA(*cozEb+ltaS*), ery^r, cam^r	This work
MDB35	MH225 carrying pVL2336-sgRNA(*ugtP-ltaA*), ery^r, cam^r	This work
MDB28	MH223 carrying pVL2336-sgRNA(*ugtP-ltaA*), ery^r, cam^r	This work
MDB36	MH224 carrying pVL2336-sgRNA(*ugtP-ltaA*), ery^r, cam^r	This work
MDB85	MH225 carrying pVL2336-sgRNA(*ltaS*), ery^r, cam^r	This work
MDB29	MH223 carrying pVL2336-sgRNA(*ltaS*), ery^r, cam^r	This work
MDB86	MH224 carrying pVL2336-sgRNA(*ltaS*), ery^r, cam^r	This work
MDB58	MDB3 carrying pRAB11-*cozEa*, cam^r	This work
MDB62	MDB3 carrying pRAB11-*cozEb*, cam^r	This work
MDB174	MDB3 carrying pRAB11, cam^r	This work
MDB360	NCTC8325-4 carrying pAF256-P_{tet}-*cozEa-smbit*/*lgbit*, cam^r	This work
MDB361	NCTC8325-4 carrying pAF256-P_{tet}-*cozEb-smbit*/*lgbit*, cam^r	This work
MDB364	NCTC8325-4 carrying pAP118-P_{tet}-*cozEa-smbit*/*cozEa-lgbit*, cam^r	This work
MDB365	NCTC8325-4 carrying pAP118-P_{tet}-*cozEa-smbit*/*cozEb-lgbit*, cam^r	This work
MDB366	NCTC8325-4 carrying pAP118-P_{tet}-*cozEa-smbit*/*ugtP-lgbit*, cam^r	This work
MDB367	NCTC8325-4 carrying pAP118-P_{tet}-*cozEa-smbit*/*ltaA-lgbit*, cam^r	This work
MDB369	NCTC8325-4 carrying pAP118-P_{tet}-*cozEb-smbit*/*cozEb-lgbit*, cam^r	This work
MDB370	NCTC8325-4 carrying pAP118-P_{tet}-*cozEb-smbit*/*ugtP-lgbit*, cam^r	This work
MDB371	NCTC8325-4 carrying pAP118-P_{tet}-*cozEb-smbit*/*ltaA-lgbit*, cam^r	This work
MK1582	NCTC8325-4, but with *gfp* fused to the 3' end of *cozEa*, spc^r	This work
MK1584	NCTC8325-4, but with *gfp* fused to the 3' end of *cozEb*, spc^r	This work
MDB77	NCTC8325-4, but with *gfp* fused to the 5' end of *ugtP*, spc^r	This work
MDB78	MDB77 carrying pLOW-dCas9_extra_*lacO*, ery^r	This work
MDB79	MDB78 carrying pCG248-sgRNA(*cozEa+cozEb*), ery^r, cam^r	This work
MDB89	MDB78 carrying pCG248-sgRNA(*cozEa*), ery^r, cam^r	This work
MDB90	MDB78 carrying pCG248-sgRNA(*cozEb*), ery^r, cam^r	This work
***S. aureus* NCTC8325**		
NCTC8325/ MDB68	MSSA lab strain	Lab collection
MDB80	NCTC8325 Δ*ugtP*, spc^r	This work
MDB69/ VL3222	NCTC8325 Δ*ltaA*, spc^r	(4)
MDB70	NCTC8325 carrying pLOW-dCas9_extra_*lacO*, ery^r	This work
MDB81	MDB80 carrying pLOW-dCas9_extra_*lacO*, ery^r	This work
MDB71	MDB69 carrying pLOW-dCas9_extra_*lacO*, ery^r	This work
MDB75	MDB70 carrying pCG248-sgRNA(*cozEa+cozEb*), ery^r, cam^r	This work
MDB84	MDB81 carrying pCG248-sgRNA(*cozEa+cozEb*), ery^r, cam^r	This work
MDB76	MDB71 carrying pCG248-sgRNA(*cozEa+cozEb*), ery^r, cam^r	This work
***S. aureus* SH1000**		
SH1000	MSSA lab strain, *rsbU*$^+$ *agr*$^+$ derivative of NCTC8325-4	(5)
E. coli		
IM08B	DH10B, Δ*dcm*, P_{help}-*hsdMS*, P_{N25}-*hsdS* (strain expressing the *S. aureus* CC8 specific methylation genes)	(6)
BL21-Gold (DE3)	BL21 derivative used for high-level protein expression	Stratagene

[a]. ery^r = erythromycin resistant, spc^r = spectinomycin resistant, and cam^r = chloramphenicol resistant.

Table S2. Plasmids used in this work.

Name	Description[a]	Reference
pLOW	Low-copy number staphylococcal shuttle vector with a IPTG inducible P*spac* promoter and *lacI* repressor, amp^r, ery^r	(7)
pLOW-*dCas9_aad9*	For IPTG inducible expression of dCas9, amp^r, spc^r	(8)
pLOW-*dCas9*_extra_*lacO*	For IPTG inducible expression of dCas9, amp^r, ery^r	(9)
pLOW-*cozEa-m(sf)gfp*	For IPTG inducible expression of CozEa with GFP fused to its C-terminal, amp^r, ery^r	(9)
pLOW-*cozEb-m(sf)gfp*	For IPTG inducible expression of CozEb with GFP fused to its C-terminal, amp^r, ery^r	(9)
pLOW-*m(sf)gfp*-SA1477	For IPTG inducible expression of SAOUHSC_1477 with GFP fused to its N-terminal, amp^r, ery^r	Lab collection
pLOW- *m(sf)gfp-ugtP*	For IPTG inducible expression of UgtP with GFP fused to its N-terminal, amp^r, ery^r	This work
pCG248	*E. coli*/*S. aureus* shuttle vector, amp^r, cam^r	(10)
pCG248-sgRNA(*cozEa*)	For constitutive expression of sgRNA(*cozEa*), amp^r, cam^r	(9)
pCG248-sgRNA(*cozEb*)	For constitutive expression of sgRNA(*cozEb*), amp^r, cam^r	(9)
pCG248-sgRNA(*cozEa*+*cozEb*)	For constitutive expression of sgRNA(*cozEa*+*cozEb*), amp^r, cam^r	(9)
pCG248-sgRNA(*cozEb*+*ugtP*-*ltaA*)	For constitutive expression of sgRNA(*cozEb*+*ugtP*-*ltaA*), amp^r, cam^r	This work
pCG248-sgRNA(*cozEb*+*ltaS*)	For constitutive expression of sgRNA(*cozEb*+*ltaS*), amp^r, cam^r	This work
pCG248-sgRNA(*luc*)	For constitutive expression of sgRNA(control), amp^r, cam^r	(9)
pVL2336	*E. coli*/*S. aureus* shuttle vector, amp^r, cam^r	(11)
pVL2336-sgRNA(*ugtP*-*ltaA*)	For constitutive expression of sgRNA(*ugtP*-*ltaA*), amp^r, cam^r	This work
pVL2336-sgRNA(*ltaS*)	For constitutive expression of sgRNA(*ltaS*), amp^r, cam^r	This work
pMAD	Thermosensitive shuttle vector for allelic replacement in Gram-positive bacteria, amp^r, ery^r	(12)
pMAD-*cozEa*::*spc*	For allelic replacement of *cozEa*, amp^r, ery^r, spc^r	(9)
pMAD-*cozEb*::*spc*	For allelic replacement of *cozEb*, amp^r, ery^r, spc^r	(9)
pMAD-*cozEa*::*cam*	For allelic replacement of *cozEa*, amp^r, ery^r, cam^r	(9)
pMAD-Δ*ugtP*::*spc*	For allelic replacement of *ugtP*, amp^r, ery^r, spc^r	This work
pMAD-*cozEa-m(sf)gfp_spc*	To GFP-tag *cozEa* in its native locus, amp^r, ery^r, spc^r	This work
pMAD-*cozEb-m(sf)gfp_spc*	To GFP-tag *cozEb* in its native locus, amp^r, ery^r, spc^r	This work
pMAD-P_{ugtP}-*m(sf)gfp-ugtP_spc*	To GFP-tag *ugtP* in a natural locus under the control of its native promotor, amp^r, ery^r, spc^r	This work
pRAB11	*E. coli*/*S. aureus* shuttle vector with an anhydrotetracycline inducible *xyl*/*tet* promoter, amp^r, cam^r	(10)
pRAB11-*cozEa*	For aTc inducible expression of CozEa, amp^r, cam^r	This work
pRAB11-*cozEb*	For aTc inducible expression of CozEa, amp^r, cam^r	This work
pAF256-P_{tet}-*hupA-smbit*/*lgbit*	Split luciferase vector encoding HupA-SmBiT and LgBiT (not part of a fusion protein) under the control of the anhydrotetracycline inducible promoter P_{tet}, used to construct negative split luciferase controls, cam^r	(13)
pAF256-P_{tet}-*cozEa-smbit*/*lgbit*	For aTc inducible expression of CozEa-SmBiT and LgBiT (not part of a fusion protein), cam^r	This work
pAF256-P_{tet}-*cozEb-smbit*/*lgbit*	For aTc inducible expression of CozEb-SmBiT and LgBiT (not part of a fusion protein), cam^r	This work

pAP118-P_{tet}-*hupA-smbit*/ *hupA-lgbit*	Split luciferase vector encoding HupA-SmBiT and HupA-LgBiT under the control of the anhydrotetracycline inducible promoter P_{tet}, used to study protein-protein interactions, cam^r	(13)
pAP118-P_{tet}-*cozEa-smbit*/ *hupA-lgbit*	For aTc inducible expression of CozEa-SmBiT and HupA-LgBiT, cam^r	This work
pAP118-P_{tet}-*cozEa-smbit*/ *cozEa-lgbit*	For aTc inducible expression of CozEa-SmBiT and CozEa-LgBiT, cam^r	This work
pAP118-P_{tet}-*cozEa-smbit*/ *cozEb-lgbit*	For aTc inducible expression of CozEa-SmBiT and CozEb-LgBiT, cam^r	This work
pAP118-P_{tet}-*cozEa-smbit*/ *ugtP-lgbit*	For aTc inducible expression of CozEa-SmBiT and UgtP-LgBiT, cam^r	This work
pAP118-P_{tet}-*cozEa-smbit*/ *ltaA-lgbit*	For aTc inducible expression of CozEa-SmBiT and LtaA-LgBiT, cam^r	This work
pAP118-P_{tet}-*cozEb- smbit*/ *hupA-lgbit*	For aTc inducible expression of CozEb-SmBiT and HupA-LgBiT, cam^r	This work
pAP118-P_{tet}-*cozEb-smbit*/ *cozEb-lgbit*	For aTc inducible expression of CozEb-SmBiT and CozEb-LgBiT, cam^r	This work
pAP118-P_{tet}-*cozEb-smbit*/ *ugtP-lgbit*	For aTc inducible expression of CozEb-SmBiT and UgtP-LgBiT, cam^r	This work
pAP118-P_{tet}-*cozEb-smbit*/ *ltaA-lgbit*	For aTc inducible expression of CozEb-SmBiT and LtaA-LgBiT, cam^r	This work
pCN55	*E. coli*/*S. aureus* shuttle vector, amp^r, spc^r	(14)
LtaA-pET19b	For expression and purification of LtaA, amp^r	(15)
pET19b-*cozEa*	For expression and purification of CozEa, amp^r	This study
pET19b-*cozEb*	For expression and purification of CozEb, amp^r	This study

a. amp^r = ampicillin resistant, ery^r = erythromycin resistant, spc^r = spectinomycin resistant, cam^r = chloramphenicol resistant, and kan^r = kanamycin resistant.

Table S3. Primers used in this work.

**Name**	**Sequence 5'-3'**[a]	**Description**[b]
**Primers to check for the presence of *cozEa***		
im17	ATCGGTACCCAATAAAACTAGGAGGAAATTTAAATGTTAAACAAGGTTTGGTTCC	*cozEa* F w/ KpnI RS
im18	GATGAATTCTTAGTCCTTAACATTACTGTTTG	*cozEa* R w/ EcoRI RS
**Primers to check for the deletion of *cozEa***		
mk188	ATTGGGCCCACCTAGGATC	F upstream of *cozEa* deletion
mk187	CAAACATTTATCGTTGTAATACGT	R downstream of *cozEa* deletion
**Primers to check for the presence of *cozEb***		
gs653	GATCGGATCCCAATGAAAATGAAAAGAATATAAGAAAG	*cozEb* F w/BamHI RS
gs654	GATCGAATCCTTTATTCAACTATTTTATTACTTTCTTTA	*cozEb* R
**Primers to check for the deletion of *cozEb***		
mk188	ATTGGGCCCACCTAGGATC	F upstream of *cozEb* deletion
mk195	GCGTCAACAATTACACCACAG	R downstream of *cozEb* deletion
**Primers check for the presence of pLOW plasmids**		
im218	TCTCATTCAATTCCTAGGTGG	pLOW F
im134	TGTGCTGCAAGGCGATTAAG	pLOW R
**Primers to check for the presence of pCG248/pVL2336 plasmids**		
mk26	GGATAACCGTATTACCGCCT	pCG248 F
mk25	AAATCTCGAAAATAATAGAGGGA	pCG248 R
**Primers to check for the presence of pRAB11 plasmids**		
mk23	GGATCCCCTCGAGTTCATG	pRAB11 F
mk24	GGGATGTGCTGCAAGGCGA	pRAB11 R
**Primers to check for the presence of pMAD plasmids**		
im156	AATCTAGCTAATGTTACGTTACA	pMAD F
mk177	GATGCCGCCGGAAGCGAG	pMAD R
**Primers to check for the presence of split luciferase plasmids**		
efs11	CATGCCAATACAATGTAGGC	Split luciferase F
efs12	CCATTTGGTCTGCTGATAGT	Split luciferase R
**Primers for construction of pLOW-*m(sf)gfp-ugtP***		
mdb9	ACGTGGATCCGTTACTCAAAATAAAAAGATATTGA	*ugtP* F w/ BamHI RS
mdb2	ACGTGAATTCATGATTAGCGTAATTATTTAACG	*ugtP* R w/ EcoRI RS
**Primers for construction of pMAD-P_{ugtP}-*m(sf)gfp-ugtP_spc***		
mdb3	ACCTGAATTCGGTATCGCTAGCGATGGCT	ori_up F w/ EcoRI RS
mdb4	TCGAACCCCGATGTTGTCG	ori_up R
mdb5	**CGACAACATCGGGGTTCGA**CAATATGTTTATTATACACGT	P_{ugtP} F overlapping ori_up
mdb6	**GTGAACAGCTCTTCTCCTTTTGACAT**TAATAGCCACCCTCCGTTAG	P_{ugtP} R overlapping *gfp*
mk48	ATGTCAAAAGGAGAAGAGCTGTTCAC	*gfp* F

mdb7	**GATCCTAGGTGGGCCCAAT**TTATTTAACGAAGAATCTTGCATATAAAG	*ugtP* R overlapping *spc*
mk188	ATTGGGCCCACCTAGGATC	*spc* F
mdb8	AGGTGTCGACATTGGTGGTATCGCTGTTGC	ori_down R w/ SalI RS
**Primers for construction of pMAD-Δ*ugtP*::*spc***		
mk501	CAACGCCTCGCAGTCGTCC	*ugtP*_up F
mk502	**TTTCCGTTAATCAAATTGCTCAT**TAATAGCCACCCTCCGTTAG	*ugtP*_up R overlapping *spc*
mk503	ATGAGCAATTTGATTAACGGAAA	*spc* F
mk504	CTAATTGAGAGAAGTTTCTATAG	*spc* R
mk505	**CTATAGAAACTTCTCTCAATTAG**AAAATTAAGTATGCTACACAGAC	*ugtP*_down F overlapping *spc*
mk506	ACGTGGATCCGATAGCTAAAGCGATAATCCAC	ugtP_down R w/ BamHI RS
**Primers for construction of split luciferase constructs**		
mdb72	ATCGGAGCTC*CAATAAAACTAGGAGGAAATTTAA*ATGTTAAACAAGGTTTGGTTCC	CozEa-SmBit F w/ SacI RS and RBS
mdb73	GCCCTCGAGCGTCCTTAACATTACTGTTTGCTT	CozEa-SmBit R w/ XhoI RS
mdb74	ATCGGAGCTC*CAATAAAACTAGGAGGAAATTTAA*ATGAATGAAAATGAAAAGAATATAAG	CozEb-SmBit F w/ SacI RS and RBS
mdb75	GCCCTCGAGCTTCAACTATTTTATTACTTTCTTTAA	CozEb-SmBit R w/ XhoI RS
mdb76	ATCGCGATCG*CAATAAAACTAGGAGGAAATTTAA*ATGTTAAACAAGGTTTGGTTCC	CozEa-LgBit F w/ PvuI RS and RBS
mdb77	GCCGCGGCCGCGTCCTTAACATTACTGTTTGCTT	CozEa-LgBit R w/ NotI
mdb78	ATCGCGATCG*CAATAAAACTAGGAGGAAATTTAA*ATGAATGAAAATGAAAAGAATATAAG	CozEb-LgBit F w/ PvuI RS and RBS
mdb79	GCCGCGGCCGCTTCAACTATTTTATTACTTTCTTTAA	CozEb-LgBit R w/ NotI RS
mdb80	ATCGCGATCG*CAATAAAACTAGGAGGAAATTTAA*ATGGTTACTCAAAATAAAAAGATAT	UgtP-LgBit F w/ PvuI RS and RBS
mdb81	GCCGCGGCCGCTTTAACGAAGAATCTTGCATATAA	UgtP-LgBit R and NotI RS
mdb82	ATCGCGATCG*CAATAAAACTAGGAGGAAATTTAA*ATGGAAAGGTTCCTTTATATGC	LtaA-LgBit F w/ PvuI RS and RBS
mdb83	GCCGCGGCCGCCTTAGCTTTTTCTCTATTTACTAT	LtaA-LgBit R w/ NotI RS
**Primers for construction of pRAB11-constructs**		
**i**m17	ATCGGTACC*CAATAAAACTAGGAGGAAATTTAA*ATGTTAAACAAGGTTTGGTTCC	pRAB11-*cozEa* F w/ KpnI RS and RBS
im18	GATGAATTCTTAGTCCTTAACATTACTGTTTG	pRAB11-*cozEa* R w/ EcoRI RS
im19	ATCGGTACC*CAATAAAACTAGGAGGAAATTTAA*ATGAATGAAAATGAAAAGAATATAAG	pRAB11-*cozEb* F w/ KpnI RS and RBS
im20	GATGAATTCTTATTCAACTATTTTATTACTTTCTT	pRAB11-*cozEb* R w/ EcoRI RS
**Primers for construction of pVL2336-constructs**		
SAOUHSC_00953_for	TATACGTGCTCAATGACGCTTAAA	pVL2336-sgRNA(*ugtP-ltaA*) F
SAOUHSC_00953_rev	AAACTTTAAGCGTCATTGAGCACG	pVL2336-sgRNA(*ugtP-ltaA*) R
SAOUHSC_00728_for	TATACTTCAAGGTAATCGTTATTA	pVL2336-sgRNA(*ltaS*) F

SAOUHSC_00728_rev	AAACTAATAACGATTACCTTGAAG	pVL2336-sgRNA(*ltaS*) R
**Primers for construction of pMAD-*cozEa-m(sf)gfp _spc***		
mk432	ACGTCCATGGATGTTAAACAAGGTTTGGTTCC	*cozEa* F w/ NcoI RS
mk433	**GATCCTAGGTGGGCCCAAT**TTACTTATAAAGCTCATCCATGCC	*gfp* R overlapping *spc*
mk188	ATTGGGCCCACCTAGGATC	*spc* F
mk434	ACGTGTCGACTGGGATTAGATATTCTATCCGT	*cozEa*_down R w/ SalI RS
**Primers for construction of pMAD-*cozEb-m(sf)gfp _spc***		
mk435	ACGTCCATGGATGAAAATGAAAAGAATATAAGAAAG	*cozEb* F w/ NcoI RS
mk433	**GATCCTAGGTGGGCCCAAT**TTACTTATAAAGCTCATCCATGCC	*gfp* R overlapping *spc*
mk188	ATTGGGCCCACCTAGGATC	*spc* F
mk436	ACGTGTCGACTCGGGTGGTCTAACCATTGA	*cozEb*_down R w/ SalI RS
**Primers for construction of pET19b-*cozEa* and pET19b-*cozEb***		
mk508	CGGCACTAGTCAT ATGTTAAACAAGGTTTGGTTCC	pET19b-*cozEa* F w/ SpeI RS
mk509	ACCAGGATCCTTAGTCCTTAACATTACTGTTTG	pET19b-*cozEa* R w/ BamHI RS
mk510	CGGCACTAGTCATATGAATGAAAATGAAAAGAATATAAGA	pET19b-*cozEb* F w/ SpeI RS
mk512	ACCAGGATCCTTATTCAACTATTTTATTACTTTCTT	pET19b-*cozEb* R w/ BamHI RS

a. The restriction sites are underlined, the overhangs are bolded, and the ribosomal binding sites are italicized.
b. F = forward primer, R = reverse primer, RS = restriction site, and RBS = ribosomal binding site.

SI References

1. Wood A, Irving SE, Bennison DJ, Corrigan RM. 2019. The (p)ppGpp-binding GTPase Era promotes rRNA processing and cold adaptation in *Staphylococcus aureus*. PLoS Genet 15:e1008346.
2. Fey PD, Endres JL, Yajjala VK, Widhelm TJ, Boissy RJ, Bose JL, Bayles KW. 2013. A genetic resource for rapid and comprehensive phenotype screening of nonessential *Staphylococcus aureus* genes. mBio 4:e00537-12.
3. Novick R. 1967. Properties of a cryptic high-frequency transducing phage in *Staphylococcus aureus*. Virology 33:155-66.
4. Zhang B, Liu X, Lambert E, Mas G, Hiller S, Veening JW, Perez C. 2020. Structure of a proton-dependent lipid transporter involved in lipoteichoic acids biosynthesis. Nat Struct Mol Biol 27:561-569.
5. Horsburgh MJ, Aish JL, White IJ, Shaw L, Lithgow JK, Foster SJ. 2002. σ^B modulates virulence determinant expression and stress resistance: characterization of a functional *rsbU* strain derived from *Staphylococcus aureus* 8325-4. J Bacteriol 184:5457-5467.
6. Monk IR, Tree JJ, Howden BP, Stinear TP, Foster TJ. 2015. Complete bypass of restriction systems for major *Staphylococcus aureus* lineages. mBio 6:e00308-15.
7. Liew ATF, Theis T, Jensen SO, Garcia-Lara J, Foster SJ, Firth N, Lewis PJ, Harry EJ. 2011. A simple plasmid-based system that allows rapid generation of tightly controlled gene expression in *Staphylococcus aureus*. Microbiology 157:666-676.
8. Myrbråten IS, Stamsås GA, Chan H, Angeles DM, Knutsen TM, Salehian Z, Shapaval V, Straume D, Kjos M. 2022. SmdA is a novel cell morphology determinant in *Staphylococcus aureus*. mBio 13:e03404-21.
9. Stamsås GA, Myrbråten I, Straume D, Salehian Z, Veening J-W, Håvarstein LS, Kjos M. 2018. CozEa and CozEb play overlapping and essential roles in controlling cell division in *Staphylococcus aureus*. Mol Microbiol 109:615-632.
10. Helle L, Kull M, Mayer S, Marincola G, Zelder M-E, Goerke C, Wolz C, Bertram R. 2011. Vectors for improved Tet repressor-dependent gradual gene induction or silencing in *Staphylococcus aureus*. Microbiology 157:3314-3323.
11. Liu X, Bakker Vd, Heggenhougen MV, Mårli MT, Frøynes AH, Salehian Z, Porcellato D, Angeles DM, Veening J-W, Kjos M. 2023. Genome-wide CRISPRi screens reveal the essentialome and determinants for susceptibility to dalbavancin in *Staphylococcus aureus*. bioRxiv doi:10.1101/2023.08.30.555613:2023.08.30.555613.
12. Arnaud M, Chastanet A, Debarbouille M. 2004. New vector for efficient allelic replacement in naturally nontransformable, low-GC-content, Gram-positive bacteria. Appl Environ Microbiol 70:6887-6891.
13. Oliveira Paiva AM, Friggen AH, Qin L, Douwes R, Dame RT, Smits WK. 2019. The bacterial chromatin protein HupA can remodel DNA and associates with the nucleoid in *Clostridium difficile*. J Mol Biol 431:653-672.
14. Charpentier E, Anton AI, Barry P, Alfonso B, Fang Y, Novick RP. 2004. Novel cassette-based shuttle vector system for Gram-positive bacteria. Appl Environ Microbiol 70:6076-6085.
15. Lambert E, Mehdipour AR, Schmidt A, Hummer G, Perez C. 2022. Evidence for a trap-and-flip mechanism in a proton-dependent lipid transporter. Nat Commun 13:1022.
