# Supplementary figures and images for: The function of CozE proteins is linked to lipoteichoic acid biosynthesis in Staphylococcus aureus
Source: mBio. 2024 May 17;15(6):e01157-24. doi: 10.1128/mbio.01157-24 (PMC11237490; doi:10.1128/mbio.01157-24)

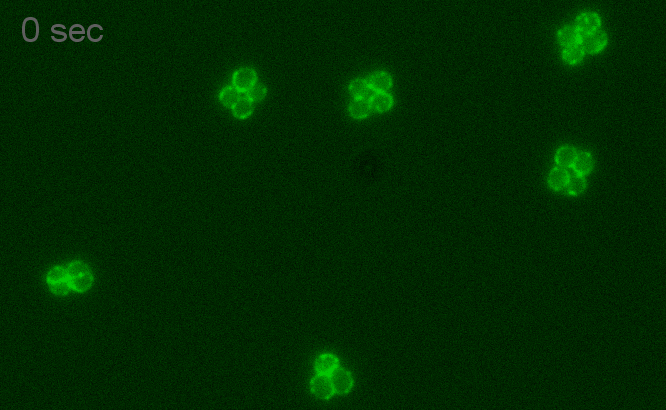

Supplement: Movie S1 — The dynamic spatiotemporal localization of GFP-tagged CozEa. [file mbio.01157-24-s0002.gif]

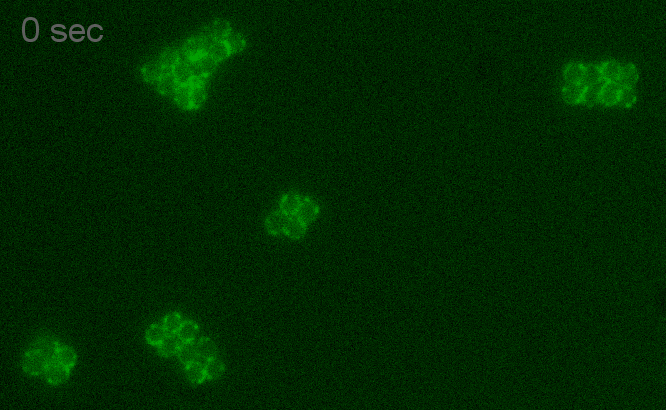

Supplement: Movie S2 — The dynamic spatiotemporal localization of GFP-tagged CozEb. [file mbio.01157-24-s0003.gif]
